# Partial purification and biochemical characterization of a new highly acidic NYSO laccase from *Alcaligenes faecalis*

**DOI:** 10.1186/s43141-020-00088-w

**Published:** 2020-11-27

**Authors:** Soad A. Abdelgalil, Ahmad R. Attia, Reyed M. Reyed, Nadia A. Soliman

**Affiliations:** 1 Bioprocess Development Department, Genetic Engineering and Biotechnology Research Institure (GEBRI), City of Scientific Research and Technological Applications (SRTA-City), New Burg El-Arab City, Alexandria, 21934 Egypt; 2 Environmental Studies Department, Institute of Graduate Studies and Research, Alexandria, Egypt

**Keywords:** Laccase, Purification, *Alcaligenes faecalis* NYSO, Decolorization

## Abstract

**Background:**

Due to the multitude industrial applications of ligninolytic enzymes, their demands are increasing. Partial purification and intensive characterization of contemporary highly acidic laccase enzyme produced by an Egyptian local isolate designated *Alcaligenes faecalis* NYSO were studied in the present investigation.

**Results:**

*Alcaligenes faecalis* NYSO laccase has been partially purified and intensively biochemically characterized. It was noticed that 40–60% ammonium sulfate saturation showed maximum activity. A protein band with an apparent molecular mass of ~ 50 kDa related to NYSO laccase was identified through SDS-PAGE and zymography. The partially purified enzyme exhibited maximum activity at 55 °C and pH suboptimal (2.5–5.0). Remarkable activation for enzyme activity was recognized after 10-min exposure to temperatures (T) 50, 60, and 70 °C; time elongation caused inactivation, where ~ 50% of activity was lost after a 7-h exposure to 60 °C.

Some metal ions Cu^2+^, Zn^2+^, Co^2+,^ Ni^2+^, Mn^2+^, Cd^2+^, Cr^2+^, and Mg^2+^ caused strong stimulation for enzyme activity, but Fe^2+^ and Hg^2+^ reduced the activity. One millimolar of chelating agents [ethylenediamine tetraacetic acid (EDTA), sodium citrate, and sodium oxalate] caused strong activation for enzyme activity. Sodium dodecyl sulfate (SDS), cysteine-HCl, dithiothreitol (DTT), β-mercaptoethanol, thioglycolic acid, and sodium azide caused strong inhibition for NYSO laccase activity even at low concentration. One millimolar of urea, imidazole, kojic acid, phenylmethylsulfonyl fluoride (PMSF), H_2_O_2_, and Triton X-100 caused activation. The partially purified NYSO laccase had decolorization activity towards different dyes such as congo red, crystal violet, methylene blue, fast green, basic fuchsin, bromophenol blue, malachite green, bromocresol purple eriochrome black T, and Coomassie Brilliant Blue R-250 with various degree of degradation. Also, it had a vast range of substrate specificity including lignin, but with high affinity towards p-anisidine.

**Conclusion:**

The promising properties of the newly studied laccase enzyme from *Alcaligenes faecalis* NYSO strain would support several industries such as textile, food, and paper and open the possibility for commercial use in water treatment. It will also open the door to new applications due to its ligninolytic properties in the near future.

## Background

In the past few years, enzymatic bioremediation has become an attractive alternative to further support the bio-treatment techniques, where the most currently available enzymes provide simpler systems than a whole organism [1]. Most xenobiotics can be submitted to enzymatic bioremediation, for example, polycyclic aromatic hydrocarbons (PAHs), polynitrated aromatic compounds, pesticides such as organochlorine insecticides, bleach-plant effluents, synthetic dyes, polymers, and wood preservatives (creosote, pentachlorophenol) [2]. Historically, the most studied enzymes in bioremediation are bacterial mono- or di-oxygenases, reductases, dehalogenases, cytochrome P450 monooxygenases, enzymes involved in lignin metabolism (such as laccases and lignin and manganese peroxidases), and bacterial phosphotriesterases [3]. From an environmental point of view, the use of enzymes instead of chemicals or microorganisms undoubtedly presents some advantages [4, 5]. Laccases (EC 1.10.3.2, benzenediol-oxygen oxidoreductase) are either mono- or multimeric copper-containing oxidases that catalyze the reduction of oxygen to water accompanied by the oxidation of phenolic and non-phenolic substrates. Laccases catalyzed the oxidation of a wide variety of organic and inorganic substrates, including mono-, di-, and polyphenols, amino phenols, methoxy phenols, aromatic amines, and ascorbate with the concomitant four-electron reduction of oxygen to water [6]. Intensive studies focused on laccases [7] because of their ability to oxidize phenolic/non-phenolic compounds as well as reduce molecular oxygen. Laccases have been found in various plants, bacteria, and also fungi; they play important roles in many cellular or microbial activities [8, 9].

Many of the industrial applications of the ligninolytic enzymes (viz, dye decolorization, phenol degradation, biobleaching, etc.) require only crude preparations of the enzyme, and in such cases, the enzyme purification is not obligatory. However, in certain cases, purified enzymes are also used for some applications [10]. The purification and enrichment of lignin-degrading enzymes such as laccases are of great interest. Moreover, ammonium sulfate fractionation is commonly used to partially purify laccases from the crude sources [11].

Due to the high industrial applicability of laccases, this study addressed the partial purification, stability characteristics of a laccase produced by *Alcaligenes faecalis* NYSO, and its decolorization activity towards different dyes.

## Methods

### Microorganism maintenance

*A. faecalis* is a Gram-negative, alkali-tolerant, rod-shaped bacterium with flagella, commonly found in soil water, and other environments, and belong to the Alcaligenaceae family. In this study, *A. faecalis* strain NYSO (KP859538) isolated from a discharged effluent of tanning and leather industry, Alexandria, Egypt, was used and identified by 16S rRNA as described previously by Abdel Galil et al. [12]. Due to its feature as alkali-tolerant, it was grown and maintained in buffered LB agar slants (glycine-NaOH, pH 8.9) [12].

### Laccase activity assay and protein estimation

The bacterial cells were grown in a previously optimized buffered medium [12], and the cells were harvested by centrifugation (6000 rpm, 4 °C, 30 min), washed with citrate-phosphate buffer (0.1 M, pH 5.0), suspended in 5 ml of the same buffer, chilled in ice, then sonicated (5 × 45 s) at high-frequency ultrasound (*HFU* ~ 20 MHz) [13]. Subsequently, the cell lysate was used for the quantitative determination of laccase activity and protein concentration after separating the cell debris by centrifugation (10,000 rpm, 4 °C, 30 min). Laccase activity has been estimated by a colorimetric method using 2,2′-Azino-bis(3-ethylbenzothiazoline-6-sulfonic acid) (ABTS, Sigma Aldrich) as described by Niku-Paavola et al. [14]. The reaction mixture, composed of 25 μl of appropriately diluted enzyme extract, 1125 μl of 0.1 M citrate-phosphate buffer pH 4.0, and 350 μl of 20 mM of ABTS substrate, was incubated after mixing at 55 °C for 10 min. One unit of enzyme activity is defined as the amount of enzyme that catalyzes the oxidization 1.0 μmol of ABTS per minute under the above assay conditions; the activity was expressed in U/L according to the following equation: activity (U/L) = ΔAbs./Δ*t* × *V*_*t*_/(*ε* × *d* × *V*_s_), where *V*_*t*_ is the total volume of the assay, 29,300 is the millimolar extinction coefficient (*ε*) of ABTS, *d* is cuvette width (cm), and *V*_s_ is the enzyme volume used in the assay.

Protein concentration was determined by Lowry et al. [15]. Bovine serum albumin (BSA, Sigma) was used for the standard curve. All enzyme assay or protein concentration measurements were expressed as the average of three reading and calculated standard deviation included according to Altman and Martin-Brand [16].

### Enzyme partial purification

Forty microliters of the crude intracellular enzyme was subjected to fractional precipitation by ammonium sulfate. Salt saturation was standardized by using different cuts (0–40, 40–60, and 60–80%) of ammonium sulfate. The protein precipitate of each fraction was collected by centrifugation (12,000 rpm, 30 min, 4 °C), then suspended in 10 ml of 0.1 M citrate-phosphate buffer, pH 5.0, and dialyzed at 4 °C against the same buffer using the dialysis bag with an exclusion limit of 12 kDa.

### SDS-polyacrylamide gel electrophoresis and zymography

SDS-PAGE was performed on 12% running non-native gels as described by Laemmli [17], and resolved proteins were visualized by Coomassie Brilliant Blue R-250 staining following standard procedures. A pre-stained protein marker of broad range (6–175 kDa, Bio Labs, New England) was used as a molecular mass marker. Laccase activity within the gel was detected as described by Sheikhi et al. [13]. After running, the gel was incubated with 2.5% Triton X-100 (Applichem) in 0.1 M citrate-phosphate buffer (pH 4.0) under conditions (room T, shaking, 30 min). Afterward, it was washed five times with 0.1 M citrate-phosphate buffer pH 4.0 and incubated with 20 mM ABTS at 55 °C till the appearance of the active band (green colored).

### Enzyme characterization

#### Temperature and pH optimum

To determine the optimum T for the partially purified NYSO laccase enzyme, the reaction was carried out at different T, ranging from 25 to 70 °C. On the other hand, to detect the optimum pH, the activity of the tested enzyme was measured at different pH values (2.5–10.0). 0.1 M of the following buffers: citrate-phosphate buffer, phosphate buffer, and glycine-sodium hydroxide buffer was used for the following specified pH values: 2.5–6.0, 7–8, and 9–10, respectively. The assay reaction was carried out at optimum T.

#### Temperature and pH stability

Thermal stability of the partially purified NYSO laccase was determined by measuring the residual enzyme activity at time intervals 1, 2, 3, 4, 5, 6, 7, 8, and 50 h, after incubating an aliquot of the enzyme at T (30–80 °C, interval 10), compared to the untreated enzyme. pH stability of the tested laccase was determined by measuring the residual enzyme activity at time intervals 1, 3, 5, and 25 h, after incubating an aliquot of the enzyme at pH 2.5, 3.0, 4.0, 5.0, and 6.0, each with the correct buffer as mentioned before. NYSO laccase activity of each sample was measured under optimal assay conditions (T 55 °C and pH 4.0) and compared to the control.

#### Thermal inactivation and storage stability

Aliquots of the partially purified enzyme solution were incubated for 10 min at 30, 40, 50, 60, 70, 80, 90, and 95^o^C, then the residual activity of the laccase was estimated compared to the untreated enzyme, while, for detection of the storage stability of the partially purified enzyme, aliquots of the enzyme solution were stored at 4.0 °C, room T (25 °C), and − 20^o^C for 46 days. The residual activity of the laccase was estimated at different time intervals for each T. NYSO laccase activity of each sample was measured under optimal assay conditions (T 55 °C and pH 4.0) and compared to the control.

#### Effects of some metal ions and some chemical agents on enzyme activity

The effect of different metal ions (Mg^2+^, Cr^2+^, Mn^2+^, Hg^2+^, Cu^2+^, Fe^2+^, Ni^2+^, Cd^2+^, Zn^2+^, and Co^2+^) on the activity of the partially purified NYSO laccase was measured under optimal conditions (T 55 °C and pH 4.0), after 10-min exposure of enzyme to different metal concentrations (1 and 10 mM). Also, the effect of some chemicals like chelators (EDTA, sodium citrate, and sodium oxalate), inhibitors (sodium azide, DTT, urea, imidazole, kojic acid, cysteine-HCl, β-mercaptoethanol, thioglycolic acid, PMSF, H_2_O_2_), and surfactants (Triton X-100, SDS) on NYSO laccase activity was carried out by pre-incubating the enzyme with the tested agent at concentrations (1 and 10 mM) for 10 min at room temperature. The residual activity was assayed at optimal conditions and compared to the untreated enzyme.

#### Enzyme decolorization potential

The decolorizing ability of the partially purified laccase was evaluated by using different synthetic dyes (0.2 g%) such as malachite green (*λ*_max_ = 616), fast green (*λ*_max_ = 625), crystal violet (*λ*_max_ = 590), congo red (*λ*_max_ = 497), eriochrome black T (*λ*_max_ = 503), bromophenol blue (*λ*_max_ = 592), methylene blue (*λ*_max_ = 665), Coomassie Brilliant Blue R-250 (*λ*_max_ = 465), bromocresol purple (*λ*_max_ = 419), and basic fuchsin (*λ*_max_ = 544). Dyes were incubated with (50 μl = ~ 84.5 Units) aliquots of the partially purified laccase solution in 0.1 M citrate-phosphate buffer (pH 4.0) containing 10 mM CuSO_4_.5H_2_O at 55 °C for 1 h. Control samples without the enzyme were processed in parallel with the tested samples. The decolorization ability of laccase was determined spectrophotometrically as the relative decrease of absorbance at each maximal absorbance wavelength of the dyes. The decolorization efficiency of laccase for each dye is shown as dye decolorization (%). The decolorization efficiency *D*% was calculated as follows: dye decolorization percentage = [(initial absorbance − final absorbance)/(initial absorbance)] × 100

Average decolorization rate = *C* × *D*% × 1000/100 × *t*, where *C* = initial concentration of dye (mg/L) and *D*% = dye decolorization (%) after time (*t*) [18].

#### Substrate specificity

Spectrophotometric measurements of substrate oxidation by the partially purified enzyme were investigated at the optimal conditions (pH 4 and T 55 °C) and different concentrations (1.0 and 20 mM) using 1.5 ml reaction mixtures containing tested substrate viz., ABTS (*ε* = 29.3 mM^−1^ cm^−1^, *λ*_max_ = 436 nm), catechol (*ε* = 26 mM^−1^ cm^−1^, *λ*_max_ = 450 nm), pyrogallol (*ε* = 35 mM^−1^ cm^−1^, *λ*_max_ = 450 nm), p-anisidine (*ε* = 1.173 mM^−1^ cm^−1^, *λ*_max_ = 350 nm), 4-nitrophenol (*ε* = 18.1 mM^−1^ cm^−1^, *λ*_max_ = 405 nm), hydroquinone (*ε* = 17.542 mM^−1^ cm^−1^, *λ*_max_ = 525 nm), 4-aminophenol (*ε* = 3.4 mM^−1^ cm^−1^, *λ*_max_ = 470 nm), p-phenylenediamine (*ε* = 14.68 mM^−1^ cm^−1^, *λ*_max_ = 525 nm), syringaldazine (*ε* = 65 mM^−1^ cm^−1^, *λ*_max_ = 530 nm), guaiacol (*ε* = 26.6 mM^−1^ cm^−1^, *λ*_max_ = 465 nm), tannic acid (*ε* = 27.200 mM^−1^ cm^−1^, *λ*_max_ = 458 nm), and vanillic acid (*ε* = 2.340 mM^−1^ cm^−1^, *λ*_max_ = 390 nm).

## Results

### Partial purification of NYSO laccase

**Fig. 1:**
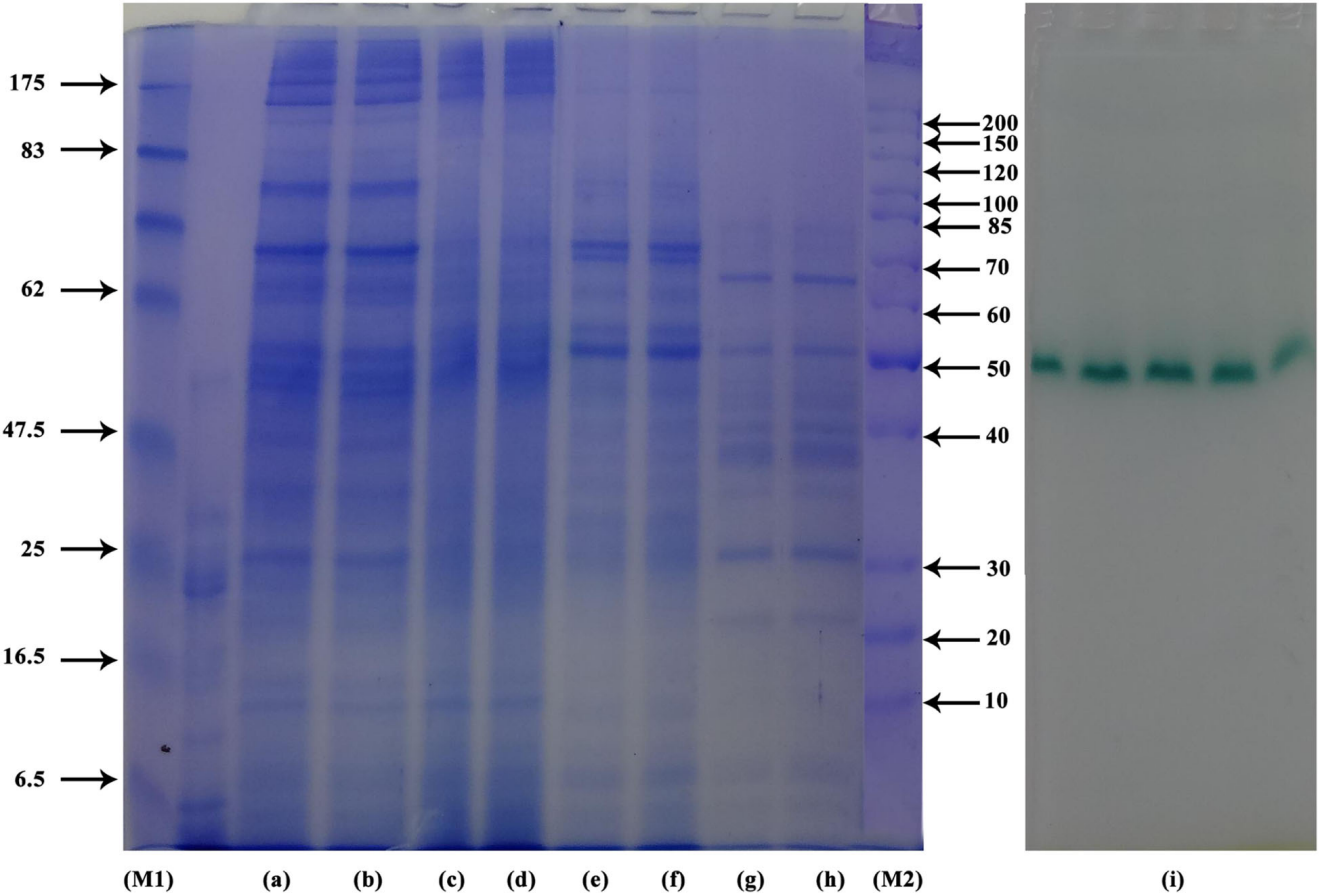
SDS-PAGE of the partially purified *A. faecalis* NYSO laccase, where M1 and M2 are the standard molecular weight markers; lanes a and b, crude NYSO laccase enzyme; lanes c and d, partially purified enzyme at 40% (NH_4_)_2_SO_4_; lanes e and f, partially purified enzyme at 60% (NH_4_)_2_SO_4_; lanes g and h, partially purified enzyme at 80% (NH_4_)_2_SO_4_; and lane i, zymography activity stain

Partial purification of the crude laccase from cell lysate of *A. faecalis* strain NYSO was carried out in three sequential steps through ammonium sulfate precipitation; the saturation 40–60% showed the maximum laccase activity (Table 1). Protein homogeneity of the partially purified NYSO laccase was checked through SDS-PAGE (Fig. 1). It was obviously recognized that there was a gradual decline of protein bands from crude extract passing to three fractionation stages. NYSO laccase activity in gel (activity staining) using ABTS substrate resulted in the appearance of an active green band at approximate molecular mass ~ 50 kDa.

**Table 1:** NYSO laccase protein fractionation using different saturation percentage of ammonium sulfate

Purification stages	Total activity^**a**^ (U)	Total protein^**a**^ (mg)
Crude enzyme	1288.7	22.005
0–40 (pellets)	204.8	18.694
40–60 (pellets)	1690.1	11.951
60–80 (pellets)	576.1	1.534

^a^Activity (U) and protein (mg) are mean of three reading

### Physicochemical characterization of the partially purified laccase

#### Effect of reaction T and pH on the enzyme activity

**Fig. 2:**
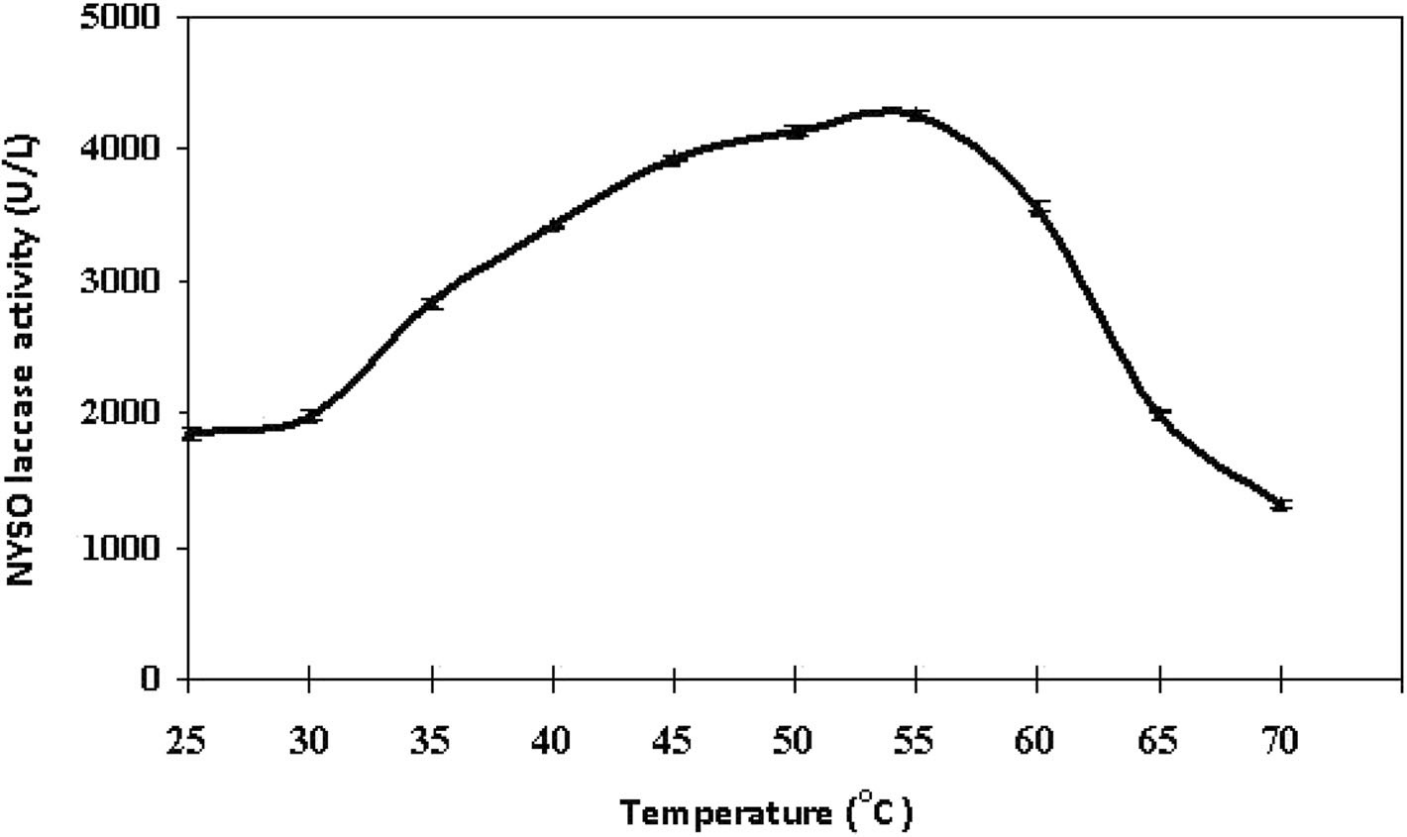
Optimum temperature for the partially purified *A. faecalis* NYSO laccase

The influence of reaction T on the enzyme activity was addressed at various incubation T (25–70 °C). The highest activity was obtained at 55 °C, while at 25 °C and 70 °C, the activity decreased to about 56% and 69%, respectively, when compared to the optimum T (Fig. 2).

**Fig. 3:**
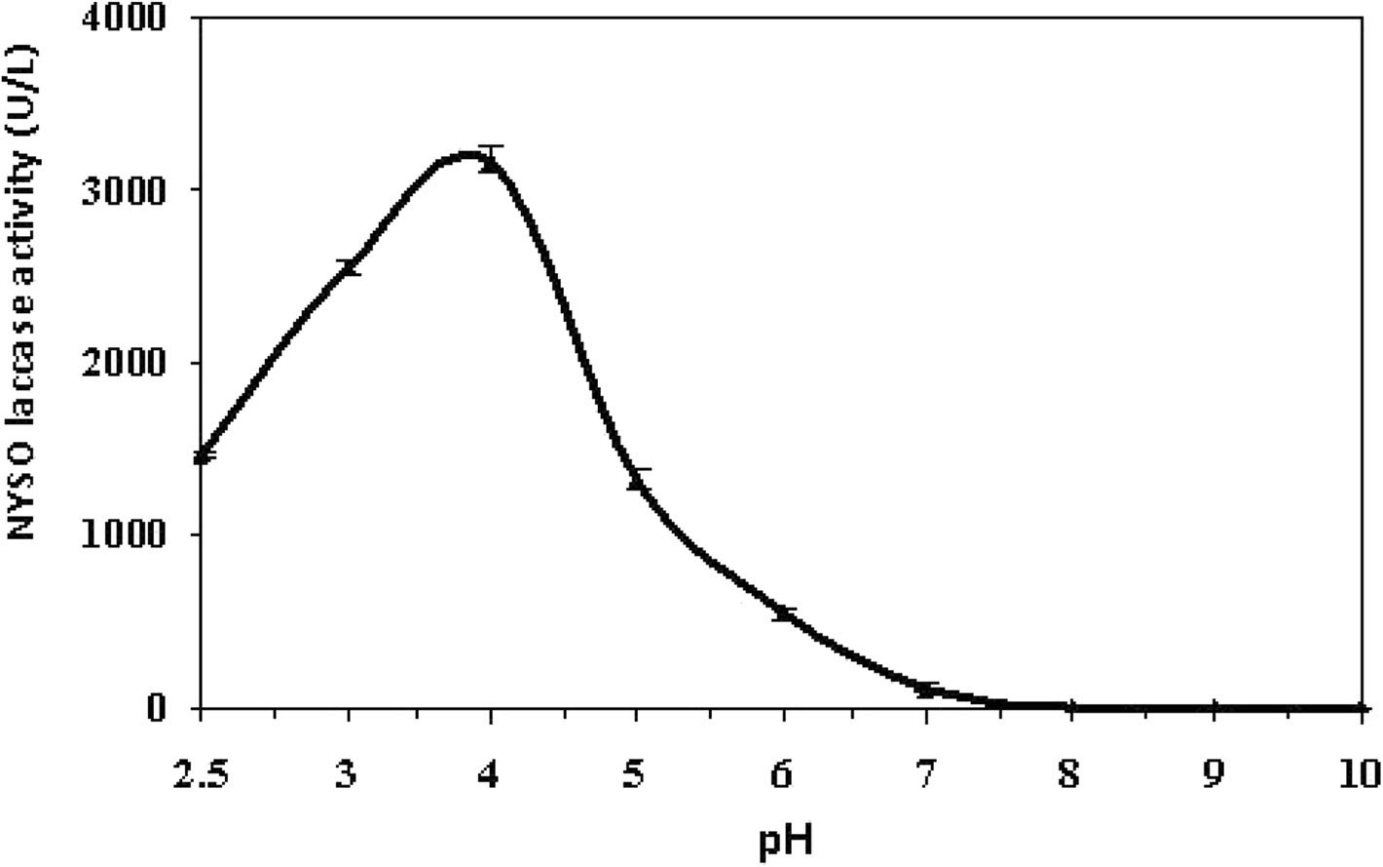
Optimum pH for the partially purified *A. faecalis* NYSO laccase

On pH profile, the partially purified enzyme works in a wide range of pH (2.5–6.0), but it had a preference to work at low pH and the optimal at 4.0 (Fig. 3). Additionally, there was a decline in activity (20%, 56.1%, and 82.8%) at pH 3, 5, and 6, respectively, when compared to pH 4.0. However, almost all activity (96.4%) was lost at neutral and no activity was detected at pH 8.0, 9.0, and 10.0.

#### Thermal and pH stability of laccase enzyme

**Fig. 4:**
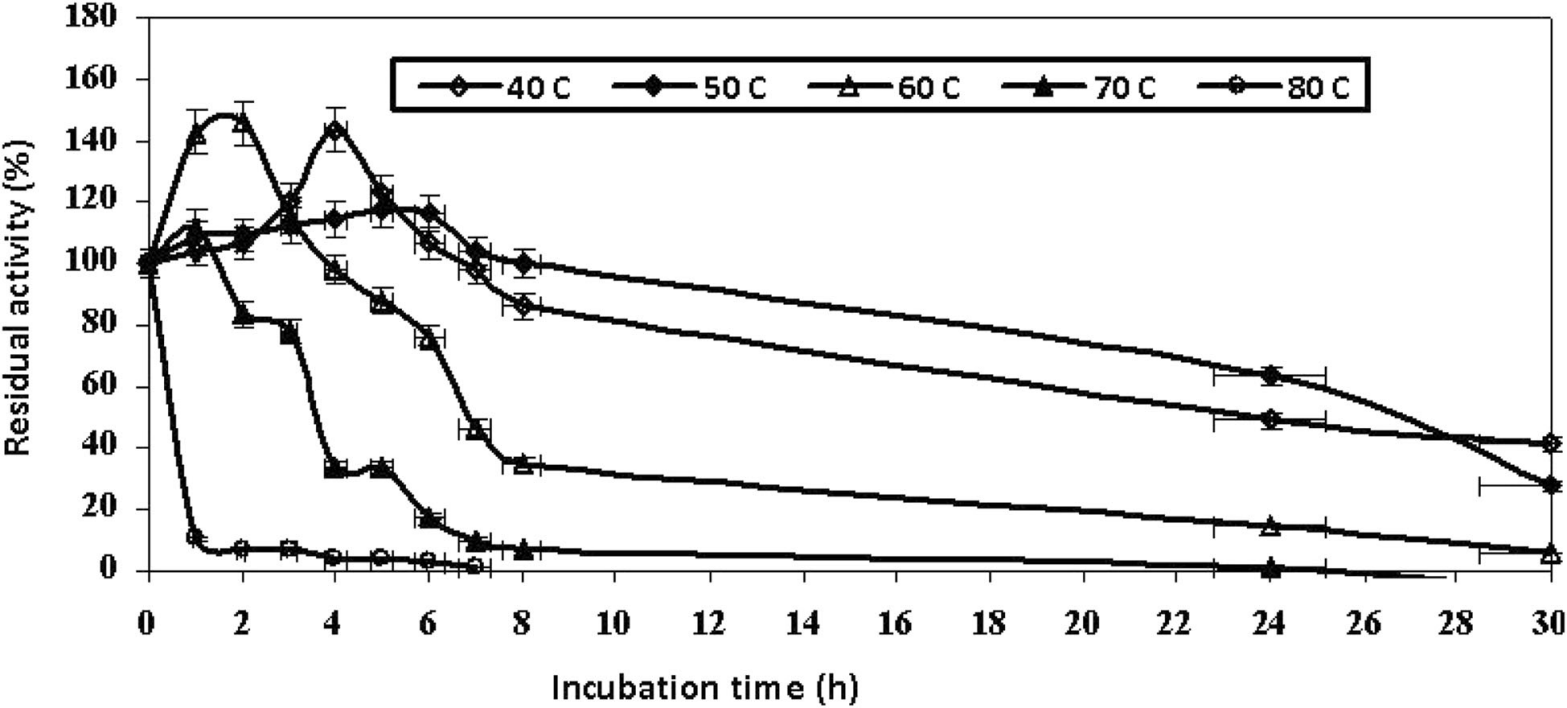
Thermal stability of the partially purified *A. faecalis* NYSO laccase

The partial purified intracellular NYSO laccase exhibited high thermal stability as shown in Fig. 4. The enzyme retained about 86.3%, 35%, and 7% of its activity after 8-h exposure to 40 °C, 60 °C, and 70 °C, respectively, when compared to the initial activity, while no loss in activity (100%) was recorded at 50 °C up to 8-h exposure.

**Fig. 5:**
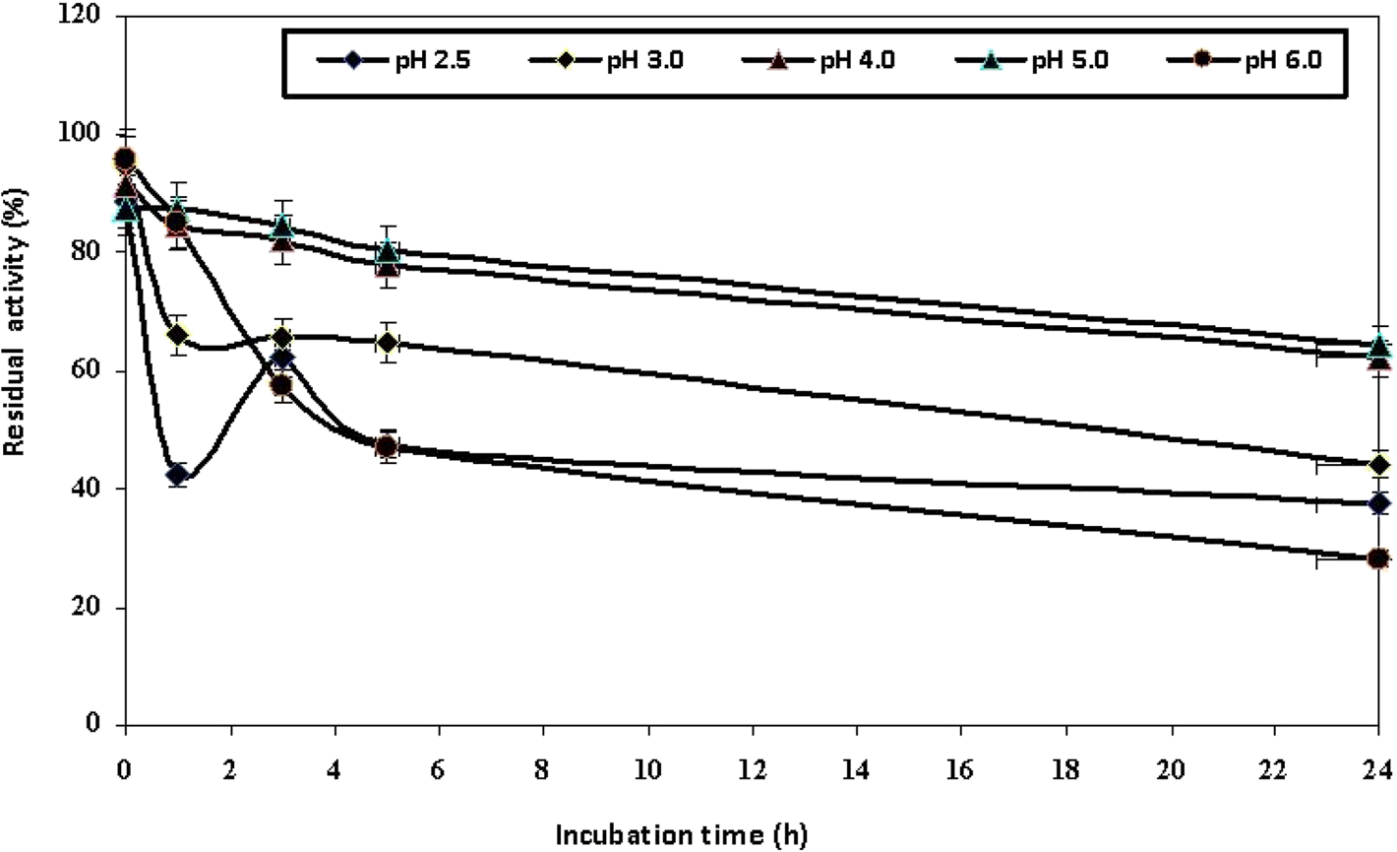
pH stability of the partially purified *A. faecalis* NYSO laccase

By studying the pH stability of the enzyme, it found that the enzyme was highly stable at pH 4 and 5 through 24-h incubation, retaining more than 92.4% and 100.3%, respectively, of the original activity. In contrast, the enzyme was unstable at pH 6.0, where about 49.1% of activity remained after 5-h incubation as shown in Fig. 5.

#### Thermal inactivation of laccase

**Fig. 6:**
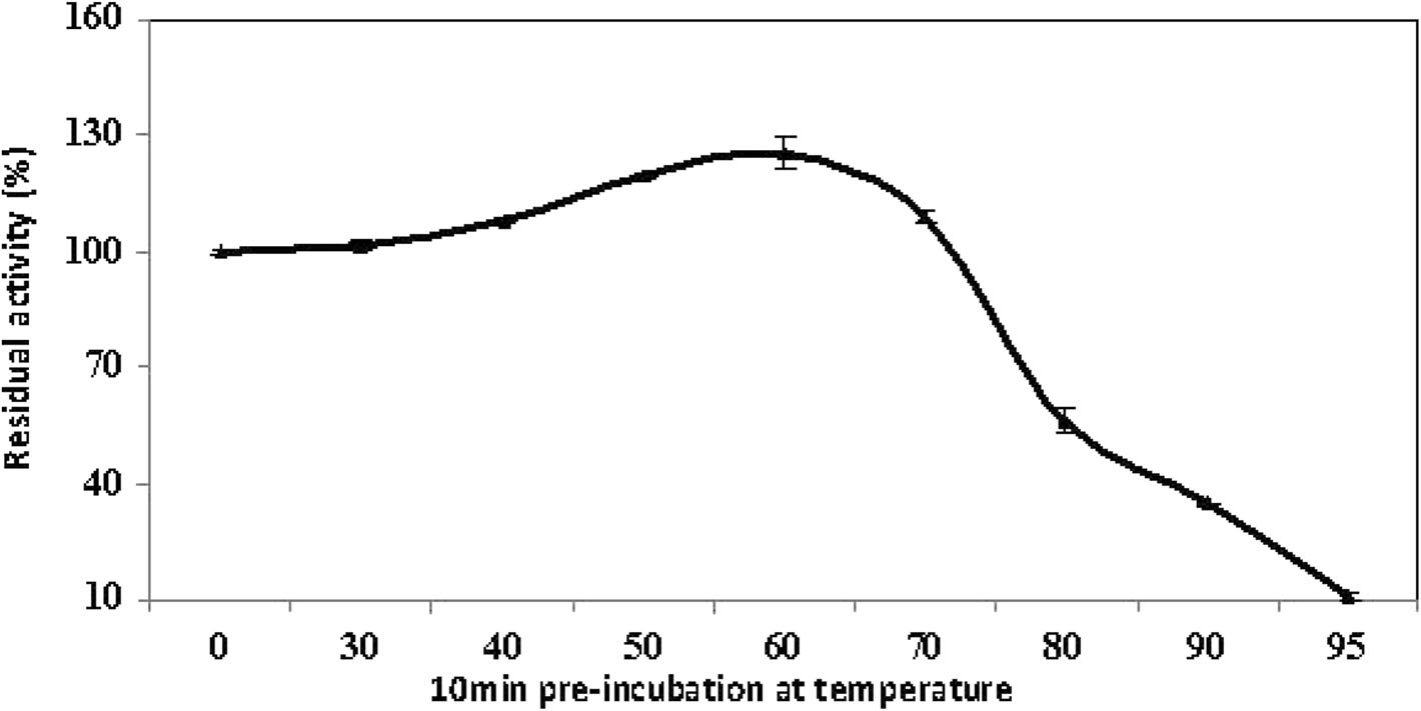
Thermal inactivation of the partially purified *A. faecalis* NYSO laccase

Figure 6 shows a gradual stimulation of the enzyme activity, when the enzyme was pre-incubated for 10 min at T 30, 40, 50, 60, and 70 °C. After that, a sharp drop in activity was observed. The enzyme showed high stability after 10-min pre-incubation at tested T up to 70 °C; however, it was noticed that there were 45% and 66% decrease upon pre-incubation at 80 and 90 °C, respectively.

**Fig. 7:**
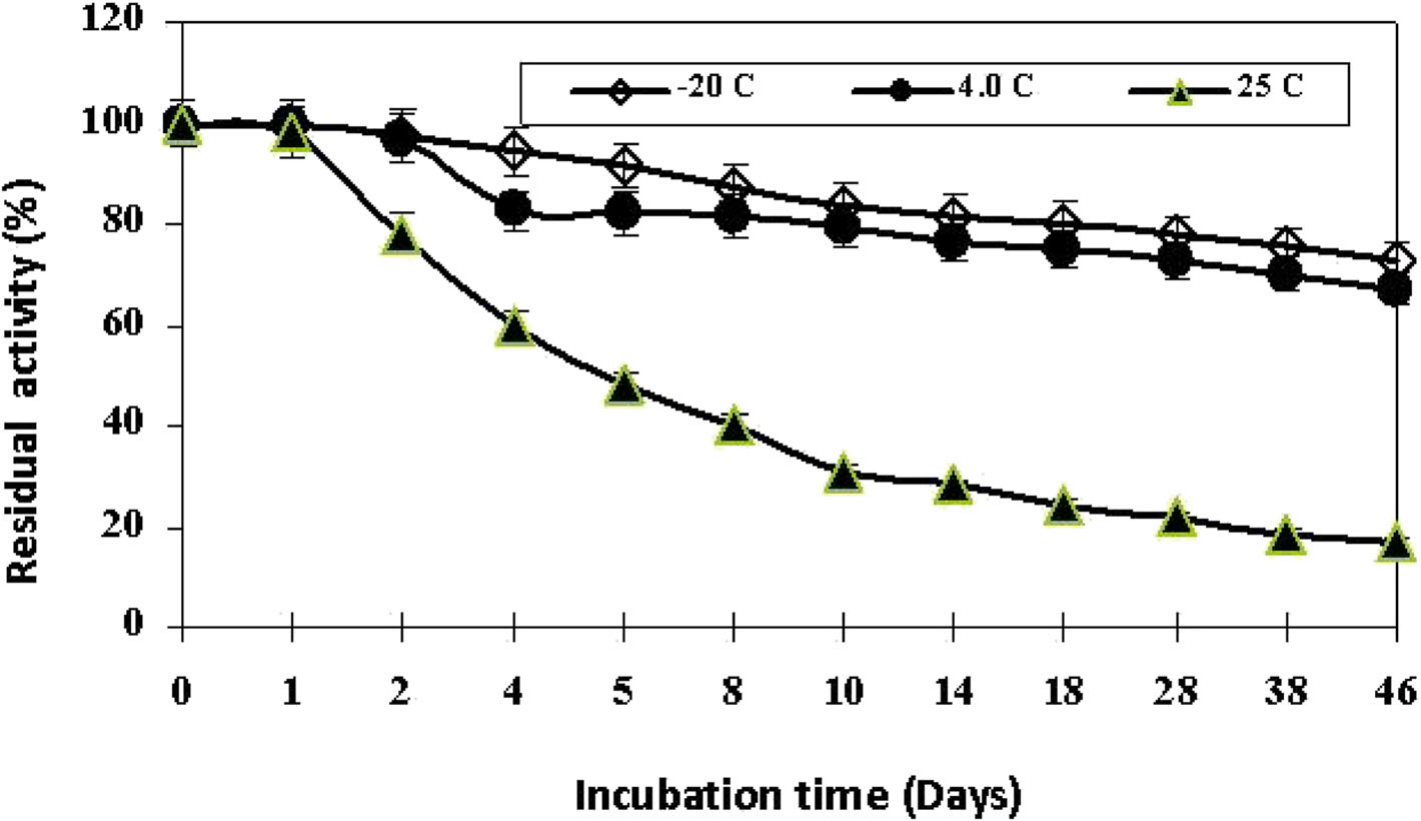
Effect of storage temperatures on the partially purified *A. faecalis* NYSO laccase

On the other hand, storage stability results of the studied enzyme explained that it was more stable at − 20 and 4 °C than room T along storage time (46 days) as shown in Fig. 7. It maintained 82% and ~ 50% of its activity after 5-day incubation at 4 °C and room T (25 °C), respectively. This means a short shelf-life for the tested partially purified laccase at room T. Consequently, after 46 days (at room T), the enzyme retained only 16.9% of its activity.

#### Effect of some metal ions and chemical reagents on laccase activity

The effect of various divalent metal ions at concentrations of 1 and 10 mM on the enzyme activity was assessed and presented in Table 2. The results showed that NYSO laccase activity was enhanced in the presence of Cu^2+^, Ni^2+^, Co^2+^, Cd^2+^, and Mn^2+^ metal ions at concentration 10 mM, in ascending order. Moreover, Zn^2+^, Cr^2+^, and Mg^2+^ metal ions at concentration 1 mM caused stimulation of activity, while lesser stimulation occurred at 10 mM (Table 2). On the other hand, both Fe^2+^ and Hg^2+^ metal ions (1 and 10 mM) caused strong inhibition for NYSO laccase activity as shown in Table 2. Also, the effects of several chelating agents and potential inhibitors on the partially purified NYSO laccase activity were investigated and reported in Table 2. Generally, the used chelating agents caused an enhancement in activity; EDTA stimulated the tested enzyme two times at 1 mM concentration, while it was strongly inhibited at 10 mM compared to the untreated enzyme sample. Sodium citrate and oxalate caused limited activation (14.7 and 3.6%) and slight inhibition (0.6 and 4.1%) at 1.0 mM and 10 mM, respectively. SDS, β-mercaptoethanol, DTT, cysteine-HCl, thioglycolic acid, and sodium azide showed an excited trend of decreasing the activity by increasing their concentration. The strongest (99.4%) was recorded by SDS, while the least (79.5) by sodium azide at 10 mM. Urea, Triton X-100, imidazole, kojic acid, PMSF, and H_2_O_2_ caused stimulation of the NYSO laccase activity by 1.57, 1.40, 1.407, 1.253, 1.062, and 1.08 times, while higher concentration (10 mM) of kojic acid, PMSF, and H_2_O_2_ caused inhibition in enzyme activity, respectively.

**Table 2:** Effect of some metal ions, chelating agents, and some inhibitors/activators on the partially purified NYSO laccase

Relative activity (%) (mean ± SD)		
**Tested**	**Metal ion concentration**	
	**1 mM**	**10 mM**
**Control**	100 ± 0	100 ± 0
MgCl_2_	157.8 ± 0.76	127.8 ± 0.75
CrCl_2_	169.2 ± 2.25	148.7 ± 1.52
MnCl_2_	140.1 ± 0.76	151.9 ± 1.70
HgCl_2_	60.1 ± 0.76	59.9 ± 0.66
CuSO_4_.5H_2_O	171.1 ± 0.89	241.8 ± 2.02
FeSO_4_.7.H_2_O	15.6 ± 0.58	10.9 ± 0.85
NiSO_4_	141.2 ± 1.10	198.5 ± 1.25
CdSO_4_	167.9 ± 1.76	166.5 ± 1.36
ZnSO4.7H_2_O	177.4 ± 2.16	171.8 ± 2.01
CoSO_4_	150.6 ± 0.65	191.3 ± 1.42
	**Chelating agent concentration**	
	**1 mM**	**10 mM**
EDTA	215.6 ± 1.25	24.6 ± 0.53
Sodium citrate	114.7 ± 0.3	99.4 ± 0.51
Sodium oxalate	103.6 ± 2.36	95.9 ± 1.79
	**Inhibitor/activator concentration**	
	**1 mM**	**10 mM**
Sodium azide	69.0 ± 0.10	20.5 ± 0.50
DTT	7.8 ± 2.02	5.5 ± 0.47
Urea	157.3 ± 2.33	127. ±1.67
Imidazole	140.7 ± 1.04	125.4 ± 1.58
Kojic acid	125.3 ± 2.0	71.1 ± 1.27
Cysteine-HCl	9.3 ± 1.10	2.1 ± 0.1
β-Mercaptoethanol	7.4 ± 0.52	4.6 ± 0.5
Thioglycolic acid	11.4 ± 1.26	9.6 ± 0.5
PMSF	106.2 ± 1.04	30.1 ± 0.6
H_2_O_2_	100.8 ± 2.25	89.5 ± 0.5
Triton X-100	140.0 ± 0.95	116.2 ± 1.31
SDS	2.9 ± 0.1	0.60 ± 0.045

#### Decolorization of dyes

Decolorization of dye by partially purified NYSO laccase was evaluated and shown in Table 3. The results indicate that the tested enzyme exhibited higher potential for decolorization of congo red, methylene blue, and fast green than crystal violet, malachite green, and eriochrome black. Likewise, the rate of decolorization reached ~ 30 and ~ 20% decrease in absorbance per hour for basic fuchsin and bromophenol blue, respectively. The resistance of Coomassie Brilliant Blue R-250 and bromocresol purple to decolorization by NYSO laccase was noticed.

**Table 3:** Decolorization potential of partially purified NYSO laccase

Tested dye	***λ***_**max**_ (nm)	Decolorization rate^**a**^ (%) (mean ± SD)	Average decolorization rate^**a**^ (mg/h) (mean ± SD)
Congo red	497	61.3 ± 2.7	122.26 ± 5.3
Crystal violet	590	47.16 ± 1.35	94.07 ± 2.69
Methylene blue	665	55.56 ± 0.63	110.82 ± 1.26
Fast green	625	54.3 ± 1.52	108.36 ± 3.04
Basic fuchsin	544	32.0 ± 3.0	63.82 ± 6.98
Bromophenol blue	592	21.3 ± 2.08	42.54 ± 4.15
Malachite green	616	36.56 ± 6.50	72.93 ± 12.9
Bromocresol purple	419	18.6 ± 3.21	37.22 ± 6.41
Eriochrome black T	503	36.0 ± 1.0	71.80 ± 1.99
Coomassie Brilliant Blue R-250	465	12.53 ± 2.50	24.99 ± 4.98

^a^Values represent mean ± SD

#### Substrate specificity

The results explained that the partially purified laccase showed broad substrate specificity, since all tested substrates were oxidized at different rates. The enzyme exhibited the highest activity with p-anisidine, while it showed a limited specificity towards syringaldazine. As reported in Table 4, the enzyme has the ability to oxidize tannic acid, vanillic acid, and pyrogallol and it showed a high affinity towards the ortho-substituted catechol among the tested substituted phenols. Lastly, the catalytic efficiency of the currently tested laccase towards phenolic substrates could be ordered as follows: p-anisidine > vanillic acid > p-phenylenediamine > 4-aminophenol > 4-nitrophenol > tannic acid > hydroquinone > ABTS > catechol > guaiacol > pyrogallol > syringaldazine.

**Table 4:** Substrate specificity of the partially purified NYSO laccase

Substrates	***λ***_**max**_ (nm)	Extinction coefficient ***ɛ*** (mM^**−1**^ cm^**−1**^)	Activity^a^ (U/L) (mean ± SD)	
			1 mM	20 mM
ABTS	436	29.3	1300 ± 9.02	7271 ± 6.98
Catechol	450	26	922 ± 8.49	4378 ± 5.77
Pyrogallol	450	35	785 ± 4.11	3336 ± 4.97
p-anisidine	350	1.173	26546 ± 108.7	83847 ± 50.57
4-nitrophenol	405	18.1	4270 ± 8.16	7920 ± 8.16
Hydroquinone	525	17.542	1318 ± 4.05	2585 ± 8.61
4-aminophenol	470	3.4	7074 ± 8.99	26390 ± 69.76
p.phylenediamine	525	14.68	11512 ± 15.14	19250 ± 122.5
Syringaldazine	530	65	744 ± 11.43	2179 ± 3.09
Guaiacol	465	26.6	801 ± 7.25	2446 ± 11.05
Tannic acid	458	27.200	1415 ± 14.72	3340 ± 4.9
Vanillic acid	390	2.340	14606 ± 76.54	26786 ± 84.01

^a^Values represent mean ± SD

## Discussion

Laccases have been mostly isolated and characterized from plants and fungi, and only fungal laccases are currently used in biotechnological applications [19]. Unfortunately, these enzymes usually work efficiently only under mild acidic conditions (pH 4–6), whereas the temperature range (30–55 °C) for catalytic activity is suboptimal. In contrast, little is known about bacterial laccases, which have a broad range of substrate specificity useful for industrial applications [19]. As a result, this study listens carefully to the partial purification and biochemical characterization of *A. faecalis* strain NYSO laccase. Initially, such bacterial species is first discovered in feces and able to degrade urea, creating ammonia which increases the pH of the environment [20, 21]. Thus, it is categorized as alkali-tolerant but it maintains a neutral pH in its cytosol to prevent the damaging or denaturing of its charged species and macromolecules [22]. Through the purification procedure for NYSO laccase produced by *A. faecalis* local isolate, it was observed that 40–60% saturation exhibited the foremost activity. This result is consistent with that attained by Singh et al. [23]. They tested different cuts (0–40, 40–60, and 60–80%) of ammonium sulfate saturation for partial purification of laccase from alkali-tolerant γ*-proteobacterium* JB, where 40–60% saturation showed the maximum laccase activity (> 60%). Moreover, activity staining of SDS-PAGE gels for NYSO laccase exhibited a single band at proximate molecular mass ~ 50 kDa. It is nearly close to *Serratia marcescens* (MTCC 4822) laccase (53 kDa) [24], but lower than the molecular mass of laccase recently obtained from *A. faecalis* (LAC1) (71 kDa) [25] and *Aquisalibacillus elongates* (75 kDa) [26]. Single band on SDS-PAGE concluded that NYSO is a monoglycoprotein, and the appearance of a colored band is due to oxidation of ABTS substrate.

Interestingly, the studied enzyme showed unlikely promising characteristic features; it can work under high acidic conditions [2.5–5; optimal at pH 4.0] and at relatively high T [45–60 °C; optimal at 55 °C]. Also, it is more stable against thermal denaturation and has high thermal stability and a long half-life when T is below 70 °C (7 h half-life at 60 °C). Most of the reported bacterial laccase showed preference to work optimally at nearly similar T and pH [27–29], but no reported bacterial laccase could work under such a highly acidic condition so far. Unlikewise, LAC1 laccase from *A. faecalis* works optimally at pH 8.0 [25]. Thermal stability for some reported laccases [29, 30] explained that the laccase activity of *Bacillus vallismortis* was accelerated after pre-incubation at 70 and 80 °C, this finding matching with the present study. Wang et al. [31] noticed that the spore-bound laccase of *Bacillus subtilis* WD23 exhibited high thermal stability (2.5 h half-life at 80 °C).

Furthermore, the investigated NYSO laccase exhibited a high pH stability, especially in acidic condition (pH 4–5) for 24-h incubation. In contrast, the enzyme was unstable at pH 6.0 where about 49.1% of activity remained after 5.0-h incubation. These results are in agreement with the results obtained by other investigators [27, 31]. The highest storage stability of the studied laccase was found at − 20 °C and 4.0 °C for a long time (up to 46 days), while at 25 °C, the enzyme activity decreased to half after 8 days of storage. Storage stability obtained in this investigation matched the results obtained by Singh et al. [23] and Bozoglu et al. [27].

Some metals (Mg^2+^, Cr^2+^, Mn^2+^, Cu^2+^, Zn^2+^, Ni^2+^, Cd^2+^, and Co^2+^), specially Cu^2+^, caused strong stimulation for the studied enzyme, while Fe^2+^ and Hg^2+^ ions caused a reduction in activity. The high extent activation of laccase by Cu^2+^ might be caused by the filling of type-2 copper-binding sites with Cu^2+^ [32]. Furthermore, most reported laccases from different bacterial sources [33, 34] are stimulated in the presence of metal cations such as Ca^2+^, Mn^2+^, Zn^2+^, Cu^2+^, Ni^2+^, Mg^2+^, and Co^2+^ which is matching with our results, while the inhibitory effect of Fe^2+^ and Hg^2+^ on laccase activity was reported by many investigators [27, 33, 34] which is similar to our results. In contrast, Niladevi et al. [35] found that Cd^2+^, Co^2+^, and Ni^2+^ metal ions caused a reduction in *Streptomyces psammoticus* laccase activity while Fe^2+^ caused activation.

Some tested agents like EDTA, sodium citrate, imidazole, and sodium oxalate caused strong activation for NYSO laccase activity at 1 mM, but cysteine-HCl, DTT, β-mercaptoethanol, sodium azide, thioglycolic acid, and SDS caused strong inhibition even at low concentration. Similarly, Rosconi et al. [36] reported that imidazole works as an activator, while β-mercaptoethanol caused a reduction of enzyme activity, though there are controversial reports about the inhibition of laccases by chelators [29]. Moreover, some studies stated that l-cysteine, EDTA, NaN_3_, SDS, DTT, and thioglycolic acid and β-mercaptoethanol caused inhibition for some bacterial laccase [27, 29, 34], while Lu et al. reported that SDS caused stimulation for the *Streptomyces* sp. laccase enzyme [37]. Additionally, the partially purified NYSO laccase showed decolorization activity towards wide varieties of synthetic dyes which is similar to mostly studied bacterial laccases [28, 29, 31].

On substrate specificity, the enzyme exhibited the highest activity towards p-anisidine; on the other hand, it showed a limited specificity towards syringaldazine (a dimer of two molecules of 2, 6 dimethoxyphenol linked by an azide bridge); these findings were in congruence with the result obtained by Niladevi et al. [35]. However, syringaldazine was used as the oxidation substrate by other investigators [31, 38]. Niladevi et al. [35] reported that the pyrogallol was denoted as the most suitable substrate for laccase from *Streptomyces psammoticus* while vanillic acid was not efficiently oxidized; these are dissimilar to our findings. According to the current results, lower activity was noted towards guaiacol, due to laccase inactivation by reaction products [39]. However, NYSO laccase positively interacts with guaiacol in the screening experiment. Similarly, Sheikhi et al. state that guaiacol is more suitable for screening laccase producer, but it is not efficient for assaying laccase [13].

## Conclusion

Laccases are one of the most widely used enzymes in industry. Thus, this study has focused on addressing the major characteristic properties of newly partially purified YNSO laccase produced by alkali-tolerant Egyptian isolate (*A. faecalis* NYSO). The theme chosen for this study and noted promising characteristics of the studied enzyme opens the door for many applications. The promising unusual features can be summarized in the following points: it is able to work in a highly acidic condition up to pH 2.5 and has high stability against thermal denaturation up to 70 °C for 1 h and high stability at pH range 4.0–5.0. The enzyme showed the highest storage stability at − 20 °C and 4.0 °C up to 46 days, while at 25 °C the enzyme activity decreased to more than half after 8 days’ storage time. Some metals (Mg^+2^, Cr^+2^, Mn^+2^, Cu^+2^, Zn^+2^, Ni^+2^, Cd^+2^, and Co^+2^) caused strong stimulation for the studied NYSO enzyme, while Fe^+2^ and Hg^+2^ ions caused a reduction of the activity. Also, chelating agents (EDTA, sodium citrate, and sodium oxalate) caused strong activation for enzyme activity at 1 mM. Cysteine-HCl, DTT, β-mercaptoethanol, sodium azide, thioglycolic acid, and SDS caused strong inhibition for NYSO laccase even at low concentrations. Additionally, the partially purified studied enzyme showed decolorization activity towards wide variants of synthetic dyes. The enzyme exhibited the highest activity with p-anisidine, and its catalytic efficiency of phenolic substrates could be ordered as follows: p-anisidine > vanillic acid > p-phenylenediamine > 4-aminophenol > 4-nitrophenol > tannic acid > hydroquinone > ABTS > catechol > guaiacol > pyrogallol > syringaldazine. The aforementioned promising features of NYSO laccase make it suitable for various industrial processes, decolorization of industrial effluent, and wastewater treatment.

## Data Availability

All data generated or analyzed during this study are included in this published article.

## Abbreviations

*A.*: *Alcaligenes*
EDTA: Ethylenediamine tetraacetic acid
DTT: Dithiothreitol
SDS-PAGE: Sodium dodecyl sulfate-polyacrylamide gel electrophoresis
PMSF: Phenylmethylsulfonyl fluoride
PAHs: Polycyclic aromatic hydrocarbons
ABTS: 2,2′-Azino-bis(3-ethylbenzothiazoline-6-sulfonic acid)
BSA: Bovine serum albumin
T: Temperature
